# Human β‐Cell Dedifferentiation and Diabetes

**DOI:** 10.1111/1753-0407.70280

**Published:** 2026-10-11

**Authors:** Jiajun Sun, Jinsook Son, Qidi Wang

**Affiliations:** ^1^ Department of Endocrine and Metabolic Diseases Shanghai Institute of Endocrine and Metabolic Diseases, Ruijin Hospital, Shanghai Jiao Tong University School of Medicine Shanghai China; ^2^ National Clinical Research Center for Endocrine and Metabolic Diseases, Key Laboratory for Endocrine and Metabolic Diseases of the National Health Commission Shanghai Key Laboratory for Endocrine Tumor, Ruijin Hospital, Shanghai Jiao Tong University School of Medicine Shanghai China; ^3^ Weill Center for Metabolic Health, Division of Endocrinology, Diabetes and Metabolism, Department of Medicine Weill Cornell Medicine New York New York USA; ^4^ Sino‐French Research Center for Life Sciences and Genomics Ruijin Hospital Affiliated to Shanghai Jiaotong University School of Medicine Shanghai China

**Keywords:** diabetes mellitus, HOMA‐β, β‐Cell dedifferentiation, β‐Cell failure, β‐Cell identity

## Abstract

β‐cell dedifferentiation in humans is considered as a mechanism underlying β‐cell failure, which is characterized by the loss of their differentiated identity and reversion to a fetal‐like state. In humans, significant increases in the hormones‐negative/Chromogranin A‐positive endocrine cells were detected in islets from T2D individuals, which were accompanied by decreased expressions of the β‐cell identity transcription factors (NKX6.1, MAFA, etc.) and reactivation of the progenitor marker, NEUROGENIN 3, and dramatic inductions of the dedifferentiation marker, ALDH1A3 and BACH2. The observation of increased dysfunctional dedifferentiated β‐cells in non‐diabetic patients with chronic pancreatitis or pancreatic ductal adenocarcinoma as well as in non‐diabetic aged subjects proposed that β‐cell dedifferentiation is not only a major mechanism of pancreatogenic diabetes and age‐related diabetes but also an initial trigger for human β‐cell failure before hyperglycemia occurs. Indeed, the finding that the ratio of dedifferentiated β‐cells is inversely related to HOMA‐β and 2h‐C Peptide in subjects without a diabetes history further supports the contribution of β‐cell dedifferentiation to β‐cell failure in humans. Considerable efforts have been made and are still required to identify targets and pathways (in)dependent of hyperglycemia that trigger dedifferentiation in human β‐cells. These findings hold promise for the development of effective therapeutic strategies aimed at preventing or reversing this process, thereby maintaining or restoring functional β‐cell mass.

## Highlights

- This article aims to briefly review β‐cell dedifferentiation in humans and to summarize promising candidates for targeting β‐cell dedifferentiation.
- The proportion of β‐cell dedifferentiation is inversely related to Homeostasis Model Assessment of β‐cell function (HOMA‐β) in humans and plays an important role in the pathogenesis of Type 2 diabetes, Type 3c diabetes, and age‐related diabetes.
- A deeper understanding of human β‐cell dedifferentiation and its underlying mechanism may help us to find therapeutic measures to reverse diabetes.

## Graphical abstract

Human β‐cell dedifferentiation is inversely associated with HOMA‐β and occurs in Type 2 diabetes, Type 3c diabetes, and age‐related diabetes, potentially before hyperglycemia develops. Progressive loss of β‐cell identity is accompanied by dedifferentiation and transdifferentiation, suggesting that reversing these cell‐state changes may help restore functional β‐cell mass.

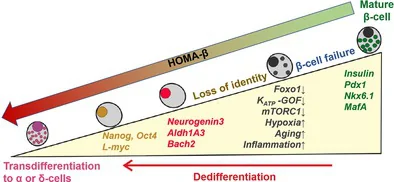

## 1 Introduction

Diabetes mellitus (DM) is a metabolic disorder characterized by an absolute or relative deficit in insulin production by β‐cells, resulting in numerous long‐term complications and organ damage [1]. A key characteristic of Type 2 diabetes (T2D) is the progressive decline in functional β‐cell mass (20%–65%) [2] eventually leading to insufficient insulin release to meet metabolic demands [3, 4, 5, 6, 7]. Elevated apoptosis has been widely recognized as a primary contributor to reduced β‐cell mass in both rodent [8, 9, 10, 11, 12, 13] and human T2D [14, 15]. However, recent studies have suggested that the limited proliferation of β‐cells or increased apoptosis in both humans [16, 17] and mouse models [18, 19] do not fully account for the progressive decrease in β‐cell mass observed in diabetes. This indicates that alternative mechanisms may contribute to β‐cell failure.

Increasing evidence underlines the importance of β‐cell dedifferentiation as a mechanism of β‐cell failure via a process involving loss of differentiated identity, reversion to a fetal‐like state, and expressing other islet hormones [18]. Loss of β‐cell identity was accompanied by concomitant increases in disallowed genes and progenitor markers, which are normally suppressed in healthy mature β‐cells [20]. The process of β‐cell dedifferentiation was first reported in β‐cell specific *FoxO1*‐deficient mice upon aging and multiparous challenges [18], and was later observed in different rodent models of obesity and T2D [19, 21]. Multiple studies found that transcription factors such as *MafA*, *Pdx1*, *Nkx6.1*, *NeuroD1* and *Islet‐1* [22, 23, 24, 25, 26, 27], cellular signals such as thyroid hormone [28], mTORC1 signaling [29, 30] and nuclear receptor ERRγ [31] were crucial for maintenance of β‐cell differentiated state and functional maturation [32]. In β‐cells, chronic hyperglycemia has been regarded as a trigger for dedifferentiation [20], as β‐cell dedifferentiation and reprogramming appeared to be reversible upon normalization of glucose levels following a short period of intensive insulin therapy in K_ATP_‐GOF mouse [19]. However, there might also exist glucotoxicity‐independent pathways that drive β‐cell to dedifferentiate under certain conditions including lipotoxicity [33, 34], oxidative stress [35, 36], endoplasmic reticulum (ER) stress [37, 38, 39], inflammation [40, 41], hypoxia [42, 43, 44] and viral infection [45]. Lineage tracing on diabetic β‐cell specific Raptor‐deficient mice showed that loss of β‐cell identity and acquisition of α‐cell features could not be entirely reversible upon glucose normalization in mutant mice [29, 30]. These studies provide insights into the molecular nature of dedifferentiation processes which require both glucotoxicity‐dependent and ‐independent pathways, and thus by translating β‐cell default biology into mechanism‐based interventions might aid to reverse the progression of T2D.

## 2 Human β‐Cell Dedifferentiation and Type 2 Diabetes

The clinical relevance is important and whether dedifferentiation involves β‐cell failure in humans needs to be further investigated. Cinti et al. demonstrated a significant increase in hormones‐negative endocrine cells in the pancreas from T2D individuals (16.8% vs. 6.5% in non‐diabetic controls) [46]. This feature was accompanied by decreased expression of the β‐cell identity transcription factors NKX6.1, MAFA and FOXO1, and a dramatic induction of the dedifferentiation marker aldehyde dehydrogenase 1 family member A3 (ALDH1A3) [46]. Moreover, the ectopic expression of FOXO1 and NKX6.1 in glucagon‐ or somatostatin‐immunoreactive cells indicated that dedifferentiated β‐cells reprogrammed to α‐ or δ‐like cells during the course of T2D [46]. Due to differences in race, age, BMI, diabetes duration, utilization of antidiabetic drugs and pancreas tissue preservation, several groups found that the ratios of dedifferentiated β‐cells in T2D islets varied from 10% to 25% [46, 47, 48], but the values were always several times higher than their non‐diabetic controls (4%~6.5%) [46, 47, 48]. Even in T2D patients with adequate glucose control (FBG < 8.3 mmol/L), a substantial portion of dedifferentiated endocrine cells (10.0% vs. 3.6% in non‐diabetic controls) was still detected [48] (Figure 1). The ratio of dedifferentiated β‐cells was positively related to diabetes duration and progression [47, 48]. Amo‐Shiinok et al. sub‐classified the patients with T2D into early‐ and advanced‐stages and found the proportion of hormones‐negative/Chromogranin A‐positive endocrine cells gradually rose, eventually reaching 25% ± 7% in advanced‐DM, supporting that islet remodeling with dedifferentiation is the underlying mechanism of β‐cell failure in the long‐standing progression of T2D [47].

**FIGURE 1:**
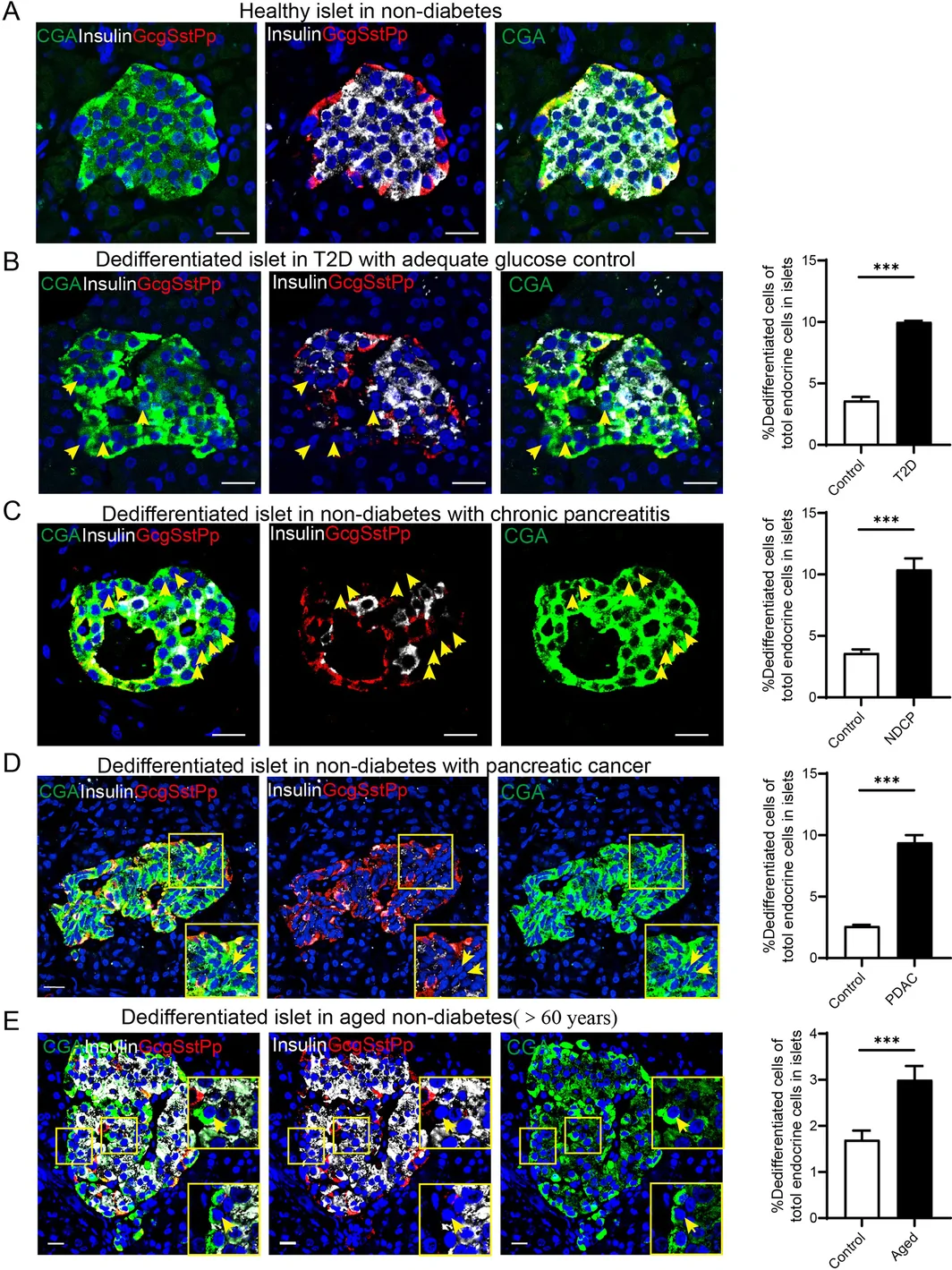
Representative immunofluorescence images of dedifferentiated islets in non‐diabetes (A), T2D with adequate glucose control (B), non‐diabetes with chronic pancreatitis (C), non‐diabetes with pancreatic cancer (D), and aged non‐diabetes (> 60 years) (E). Human islets immunostained with insulin (white), endocrine cocktail (Gcg/Sst/Pp, red), CGA (green), and DAPI (blue) are shown. Yellow arrows indicate dedifferentiated cells (endocrine hormone‐negative/CGA‐positive). The ratios of dedifferentiated cells were determined: ***p < 0.001. *Source:* Adapted from our previously published figures in 2019 JCEM [48], 2020 JCEM [49], and 2022 JCEM [50].

## 3 Human β‐Cell Dedifferentiation and HOMA‐β Ratio

To establish the direct link between β‐cell dedifferentiation and HOMA‐β values in human, Kang et al. reported that the ratios of β‐cell dedifferentiation in subjects without diabetes history were inversely correlated with their HOMA‐β values and 2 h C‐peptide levels measured 1 week before pancreatectomy [51]. Multivariate analysis showed that HOMA‐β, rather than BMI or HbA1c%, was independently and negatively correlated with the percentage of dedifferentiated cells. The pancreatic islets from HOMA‐β^low^ (< 100%) subjects were marked in situ as (1) increased β‐cell dedifferentiation ratio (8.4% vs. 4.1%), (2) induction of ALDH1A3 expression, and (3) lower expression of β‐cell functional markers GLUT2 and UCN3 [51]. The findings suggested that β‐cell dedifferentiation preceded diabetes onset and contributed to β‐cell dysfunction. Another key finding was the identification of BACH2, whose expression was enriched in HOMA‐β^low^ subjects: the percentage of BACH2^+^ β cells was nearly two‐fold higher than that in the HOMA‐β^high^ group (9.2% vs. 5.6%) [51]. BACH2 is a key regulator of cell state transitions in T2D, whose inhibition has been shown to improve β‐cell function in diabetic mice and human islets [52]. Both our [51] and Son's findings [52] underscored the critical role of Bach2 in β‐cell dedifferentiation leading to T2D and highlighted BACH2 as one of the potential therapeutic targets. Future research should focus on elucidating the molecular mechanisms underlying dedifferentiation and exploring therapeutic strategies to reverse this process, potentially leading to disease modification in T2D.

## 4 Human β‐Cell Dedifferentiation and Type 3c Diabetes

Pancreatogenic diabetes (Type 3c diabetes) can be caused by exocrine pancreatic diseases, including chronic pancreatitis and pancreatic ductal adenocarcinoma (PDAC) [53]. In chronic pancreatitis, varying degrees of exocrine inflammation were accompanied by reduced expression of the β‐cell‐specific transcription factor PDX1 [54], and 30%–50% of patients with chronic pancreatitis will eventually become diabetic during disease progression [55, 56, 57]. In non‐diabetic subjects with chronic pancreatitis, Sun et al. found a significant rise in dedifferentiated cells (~10.4%) in islets and the dedifferentiated cells formed non‐hormone “empty regions” (Figure 1) with ALDH1A3 expression [48]. This finding was consistent with the observations that proinflammatory cytokines such as interleukin 1β (IL‐1β), interleukin 6 (IL‐6), and tumor necrosis factor (TNF) could down‐regulate β‐cell‐specific transcription factors (*Pdx1*, *Nkx6.1* and *FoxO1*) [40], indicating inflammation might cause β‐cells to lose their identity.

In addition to chronic pancreatitis, inflammation has also emerged as a hallmark of pancreatic cancer [58], which has been associated with new‐onset of diabetes [59, 60, 61]. In non‐diabetic patients with pancreatic ductal adenocarcinoma (PDAC), the paraneoplastic inflammation also increased the proportion of dedifferentiated β‐cells and the PDAC islets were dysfunctional before glycemic levels rose (Figure 1) [49]. When the intact islets were exposed to conditioned medium of the human pancreatic adenocarcinoma cell line PANC‐1 for 24 h, they exhibited defects in glucose‐stimulated insulin secretion (GSIS) [49] and lost β‐cell identity, with decreased expressions of MAFA, PDX1, UCN3, and GLUT2, and increased expressions of progenitor cell markers, including NGN3 [62], OCT4 [18], and SOX9 [63]. Moreover, Qin et al. demonstrated that VNN1, secreted by pancreatic cancer cells, exacerbated β‐cell dysfunction and dedifferentiation in paraneoplastic islets through oxidative stress [64].

Taken together, data from nondiabetic patients with either chronic pancreatitis or PDCA not only propose that β‐cell dedifferentiation is a major cause of Type 3c diabetes, but also support that β‐cell dedifferentiation can serve as an initial trigger for human β‐cell failure, preceding the rise in blood glucose level.

## 5 Human β‐Cell Dedifferentiation and Age‐Related Diabetes

T2D is an age‐related disease, and the incidence and prevalence of T2D increase with age [65, 66, 67, 68, 69]. Age‐related deterioration in glucose tolerance is attributed to reduced GSIS [70, 71, 72, 73] and/or decreased insulin sensitivity in peripheral tissues [74, 75]. Early studies have suggested that insulin resistance increases with age due to increased adiposity and reduced lean muscle mass [76, 77, 78, 79, 80, 81]; however, these factors alone do not account for age‐related glucose intolerance [82]. A considerable number of studies in humans consistently found an age‐dependent decline of β‐cell function, independent of insulin resistance [72, 75, 83]. A recent study revealed that the gene profiles in senescent rodent β‐cells were similar to those of dedifferentiated dysfunctional β‐cells [84]. More importantly, senolysis (removal of senescent cells), using either a transgenic INK ATTAC model (a whole‐body FLAG‐tagged transgenic enabling B/B homodimerizer‐induced deletion of p16^Ink4a+^ cells) or oral administration of the senolytic drug ABT263, improved glucose metabolism, as well as β‐cell identity and function [85].

Recently, the single‐cell transcriptome analysis was conducted on human pancreas from eight donors spanning six decades of life: islet endocrine cells from older donors exhibited elevated levels of transcriptional noise and demonstrated increased expression of cell‐atypic hormone mRNA [86]. Such “fate drift” is emblematic of age‐dependent transcriptional instability and indicates β‐cell identity loss and reprogramming might be a novel mechanism of β‐cell failure in aging. To test whether beta cells undergo dedifferentiation during their progression to aging, Song et al. collected pancreas samples from 31 nondiabetic individuals aged 25 to 94 years, all of whom had fasting blood glucose levels within the normal range, and assessed the proportion of dedifferentiated cells in nondiabetic young (< 40 years), middle‐aged (40–60 years) and elderly (> 60 years) subjects [50]. A two‐fold increase in middle‐aged and elderly subjects was found (Figure 1), and the frequency of human dedifferentiated cells was independently and positively related to age. This was accompanied by inactivation of FOXO1 and reduced expression of UCN3 in elderly islets [50]. To date, therapies which target aging aspect of diabetes have not been well developed. Aguayo‐Mazzucato et al. have proposed senolysis improved glucose homeostasis and restored gene expression in aging mice and FACS sorted senescent human β‐cells [85]. Taking these encouraging results into account, β‐cell dedifferentiation in aging remains a promising therapeutic target for age‐related diabetes.

## 6 β‐Cell Dedifferentiation and Therapeutic Implications for Human Diabetes

Although existing drugs are very effective in controlling glycaemia, the decline in functional β‐cell mass remains inevitable [87, 88]. Sulfonylurea‐induced insulin release effectively lowered hyperglycemia, and the degranulated β‐cells in the short‐term could compensate with elevated insulin translational and synthetic activity [89, 90]; however, in the long‐term, the drug depletes insulin stores, exhausts β‐cells with impaired secretory capacity, and lacks beneficial effects on islet plasticity in diabetic mice models [91]. The dipeptidyl peptidase‐4 (DPP‐4) inhibitor, sitagliptin, and Glucagon‐like peptide‐1 (GLP‐1) analog, liraglutide, both countered β‐ to α‐cell reprogramming in diabetic rodent models induced by STZ, hydrocortisone, or high fat feeding [92]. Liraglutide in vitro treatment partially protected dedifferentiated diabetic human islets by inhibiting glucagon positive cell uprising and suppressing the induction of ALDH1A3 and NGN3 [93]. Alleviating metabolic demand on β‐cells might be a valid strategy to delay the progression of T2D [87, 94, 95, 96, 97, 98, 99, 100]. Metformin treatment was reported to rest β‐cells by suppressing insulin synthesis and release via the AMPK/mTOR pathway [101], but in the long‐term, it did not influence α−/β‐cell transdifferentiation [91]. Dapagliflozin treatment in T2D mice increased the number of insulin/glucagon double‐positive cells in areas where α‐cells appeared, suggesting a possible role in the conversion of α‐cells to β‐cells [102]; however, it needs to be validated in lineage‐tracing animal models.

In addition to oral anti‐diabetic agents, a short period of intensive insulin therapy at the time of T2D diagnosis led to rapid improvement of insulin secretion and preserved β‐cell function by allowing β‐cells to “rest” [96, 100, 103, 104]. Wang et al. demonstrated β‐cell mass and insulin content could be reversed and restored in diabetic mice following a short period of intensive insulin therapy. Lineage‐tracing analysis revealed that reversing hyperglycemia by insulin therapy led the same dedifferentiated β‐cells to re‐differentiate to mature β‐cells and reestablish antidiabetic drug‐responsivity [19]. Interestingly, Ishida et al. compared the ability of different strategies (phloridzin, rosiglitazone, insulin and pair‐feeding) on alleviating β‐cell dedifferentiation in *db/db* mice and demonstrated that β‐cell identity loss and *Aldh1a3* induction could only be reversed by paired diet, suggesting that caloric restriction can prevent, and possibly reverse, β‐cell dedifferentiation [105]. Building on this, *Aldh1a3* lineage tracing experiments using db/db mice demonstrated that pair‐feeding alone was sufficient to drive re‐differentiation of Aldh1a3^+^ dedifferentiated β‐cells back to mature β‐cells [106]. These findings underscore the role of diet in diabetes management and highlight its potential as an alternative to medication.

Meanwhile, the findings of inflammation‐triggered β‐cell dedifferentiation in non‐diabetic patients with chronic pancreatitis or pancreatic cancer provide a unifying mechanism that supersedes the debate on whether insulin resistance or β‐cell failure is to blame for pancreatogenic diabetes [53, 107, 108]. It is currently unknown whether chronic inflammation in diabetic islets [109, 110] and proinflammatory cytokines and chemokines released in T2D individuals [35] might also participate in β‐cell dedifferentiation. The finding that stearoyl CoA desaturase (SCD) reverses the process of β‐cell dedifferentiation by inhibiting inflammation in the human β‐cell line EndoC‐βH1 further emphasizes the prominent role of inflammation in dedifferentiation [111]. Interestingly, intravenous infusion of mesenchymal stem cells has been proposed to reverse the dedifferentiation of failed β‐cells via secreting IL‐1R antagonists stimulated by IL‐1β and TNF‐α in *db/db* mice [112], which reveals new insights into the treatment of diabetes.

Rodent studies have provided promising candidates for targeting β‐cell dedifferentiation, such as mature β‐cell markers (*MafA*, *Nkx6.1*, *Pdx1*, *Gck*, mTORC1), progenitor marker (*Ngn3*) [23, 25, 29, 113, 114], mitochondrial (*Cyb5r3*) [115] and excitability (*Kir6.2*) [19] genes, as well as long noncoding RNAs (*Malat1*, *Meg3*, *Kcnq1*, *Ot1*) [116, 117, 118, 119]. Gómez‐Banoy et al. presented that forced expression of *Dusp26* downregulated genes associated with β‐cell identity, which could be rescued by Adipsin/C3a via pharmacological inhibition of *Dusp26* in *db/db* mice [120]. The Accili group focused on the molecular mechanisms downstream of *FoxO1*, a multifunctional transcription factor that mediates T2D β‐cell dedifferentiation. Kim‐Muller et al. put forward that the concomitant reduction of *FoxO1* expression with activation of *Aldh1a3* was usually a harbinger of β‐cell failure. When *FoxO1* levels reached their nadir as if *FoxO* had been knocked out, not only did *Aldh1a3* increase, but the expression of genes including oxidoreductase cytochrome b5 reductase 3 (*Cyb5r3*), *Elovl6*, and *Bach2* was also significantly altered [115]. The importance of *Aldh1a3* as a candidate target was also demonstrated by Wang et al. with experiments showing that inhibition of *Aldh1a3* by microRNA‐483 could protect pancreatic β‐cells from dedifferentiation [121]. Moreover, treatment with KOTX1, a small‐molecule *Aldh1a3* inhibitor, enhanced β‐cell function, directly reducing Aldh1a3^+^ β‐cells, highlighting the therapeutic potential of targeting *Aldh1a3* in vivo [106]. Based on the evidence that dedifferentiation is associated with metabolic instability [122], Fan et al. demonstrated that *Cyb5r3*, a mitochondrial function‐related molecule, linked FoxO1‐dependent mitochondrial dysfunction to β‐cell failure but was not involved in murine β‐cell dedifferentiation [88]. More importantly, Son et al. used the functional assessment of unbiased scRNA‐Seq data from pancreatic samples of T2D patients and identified BACH2 as a strong target of the dedifferentiation process in human T2D. Treatment with a BACH inhibitor could lower hyperglycemia and increase plasma insulin concentration in diabetic mice, thereby restoring insulin secretion and reversing β‐cell failure [52].

Human pluripotent stem cell (hPSC)‐derived β cells have been widely recognized as superior humanized models to investigate β‐cell dedifferentiation and develop novel diabetes therapeutics, overcoming the limitations of traditional rodent models and primary human cadaveric islets. The landmark achievement by Pagliuca et al. established the foundational protocol for generating stem cell‐derived β cells (SC‐β cells) from hPSCs, which exhibited glucose‐stimulated insulin secretion comparable to that of adult human islets [123]. Later, various research teams optimized differentiation protocols to produce SC‐β cells with enhanced maturation and functional stability and made SC‐islet transplantation a viable cure for diabetes [124, 125, 126, 127, 128]. Recent mechanistic research on SC‐β cells identified new pathological pathways and therapeutic targets. Dieter et al. [129] employed patient‐derived SC‐β cells bearing INS mutations and found that misfolded proinsulin accumulation caused an increase in β‐cell dedifferentiation (evidenced by ALDH1A3+/NKX6.1+ cells), establishing a mechanistic link between ER stress and identity loss in permanent neonatal diabetes. It was reported that excessive zinc accumulation activates the integrated stress response and ultimately triggers the loss of β‐cell identity in SC‐β cells, while inhibition of zinc accumulation effectively safeguards SC‐β cells from identity loss and enhances their efficacy, offering a novel direction for targeted drug development [130]. Collectively, SC‐β cells not only serve as an ideal human model to uncover the molecular mechanisms of β‐cell dedifferentiation, but also act as a powerful translational platform to screen and validate novel drugs, bringing new opportunities for the treatment of diabetes.

Much more work needs to be performed to identify targets and pathways (in)dependent of hyperglycemia that trigger dedifferentiation in human β‐cells. Further exploration of single‐cell sequencing of human prediabetic and diabetic islets will have implications to deepen the fundamental understanding of the impact of dedifferentiated β‐cells and could potentially lead to the development of effective therapeutic strategies by preventing or reversing the process to maintain or regain functional β‐cell mass.

## Author Contributions

Conceptualization: Qidi Wang. Writing – original draft preparation: Qidi Wang, Jinsook Son, Jiajun Sun. Writing – review and editing: Qidi Wang and Jiajun Sun. Funding acquisition: All authors have read and agreed to the published version of the manuscript.

## Funding

This research was funded by the National Natural Science Foundation of China Grants (82470832, 82270841, 82311530702, 82300889).

## Ethics Statement

This study was approved by the Institutional Review Board of the Ruijin Hospital affiliated to Shanghai Jiao Tong University School of Medicine and was in accordance with the principles of the Declaration of Helsinki.

## Conflicts of Interest

The authors declare no conflicts of interest.

## Data Availability

Data sharing not applicable to this article as no datasets were generated or analyzed during the current study.
